# German Science and English Newspapers

**Published:** 1906-10-06

**Authors:** Hugo Lakin

**Affiliations:** London


### GERMAN SCIENCE AND ENGLISH NEWSPAPERS.

*To the Editor of* The Hospital.

Dear Sir,—Allow me to call your attention to a most clever way in which the Germans use the English daily papers for the purpose of exploiting their industrial and scientific attainments. If the German interests concerned were paying for the service, the English Press could not serve them more effectually than it is doing "without money and without price."

Recently a vivisectionist in Germany did some work on the transplanting of a dog's kidney; immediately the English daily Press devotes a good deal of space to recording this wonderful achievement which, after all, is of little, if any, practical benefit, and should it turn out to be of value, it can only be of much interest to medical men.

The only object of their frequent telegraphic accounts is to boom German science, German-made industries, and German interests in general, and the result is that the names of German scientists are perfectly familiar to the majority of English-speaking laymen in the whole world, while the names of men of equal or greater eminence in this country are comparatively unknown. I have had the good fortune to be in this country twenty days, and in that brief time I have read several accounts of the voyage of the Khedive of Egypt to German spas containing, of course, a full account of the places and the remarkable improvement of his health. I have read of a surgeon—German, of course—being called to Constantinople, a full account of his *intended* operation, and all details of his and his assistants' social reception, etc. I have read vivid accounts of the trip of a great potentate to Carlsbad, and lately, some days ago, this large telegraphic account of the doing of a Breslau vivisectionist as having something important to do with advanced surgery.

All this news originated in Berlin, and by the aid of semi-official Reuter's Agency and the English most important daily Press, is spread throughout the world. It seems to me that this indiscriminate booming and puffing-up German things scientific does an injustice to the men in this country doing similar work, and has a tendency to give Germany an importance in the minds of the public which it does not deserve.

It seems to me that the English Press is unconsciously acting in this matter in a shortsighted manner, and I hope I may be pardoned for directing attention to it.

I am, etc.,

London, October 1, 1906. Hugo Lakin, M.D.

